# Effect of Corneal Hydration on the Quality of the Femtosecond Laser Anterior Lamellar Cut

**DOI:** 10.1371/journal.pone.0098852

**Published:** 2014-06-09

**Authors:** Ossama Nada, Anca Marian, Nicolas Tran-Khanh, Michael Buschmann, Michel Podtetenev, François Vidal, Santiago Costantino, Isabelle Brunette

**Affiliations:** 1 Maisonneuve-Rosemont Hospital Research Center, Montreal, Québec, Canada; 2 Biomedical and Chemical Engineering and Groupe de Recherche en Sciences et Technologies Biomédicales, École Polytechnique, Montreal, Québec, Canada; 3 Institut National de la Recherche Scientifique – Centre Énergie Matériaux Télécommunications, Varennes, Québec, Canada; 4 Department of Ophthalmology, University of Montreal, Montreal, Québec, Canada; 5 Focus Vision Clinic, Verdun, Québec, Canada; 6 Ain Shams University, Cairo, Egypt; Johns Hopkins University, United States of America

## Abstract

The goal of this study was to assess the effect of corneal hydration on the quality of the femtosecond laser (FSL) anterior lamellar cut. The Visumax FSL was used to dissect an 8-mm-diameter corneal flap in 22 eye bank corneas showing various levels of hydration. The intended ablation depth was 220 µm in all eyes, which corresponded to the maximal depth available with this laser. After the cut, the achieved ablation depth was measured using optical coherence tomography images, flap separability was assessed by measuring the mean force generated to detach the flap, and stromal bed roughness was assessed by measuring the Haralick contrast level on the 1000× scanning electron microscopy images of the ablated surfaces. The preoperative central corneal thickness ranged from 547 to 1104 µm (mean ± SEM: 833±30 µm). A negative correlation was found between the level of corneal hydration and the ablation depth measured in the mid-peripheral cornea (r = −0.626, p = 0.003), the ablation being more superficial in more edematous corneas. The Haralick contrast also tended to increase as a function of corneal hydration (r = 0.416, p = 0.061), suggesting that laser ablation in edematous corneas results in rougher stromal surfaces. These results support the hypothesis that the quality of the FSL lamellar cut decreases as the level of corneal hydration increases. Although FSL is still considered in the field as the tool of the future for corneal dissection, a better understanding of the limits of this tool will be needed before it can replace manual or automated stromal dissection techniques in hydrated corneas.

## Introduction

Commercial femtosecond lasers (FSLs) in ophthalmology have allowed major achievements in corneal refractive surgery. They have greatly improved dissection of the anterior lamellar flap in LASIK surgery [1], with smoother ablation surface [2], greater flap uniformity [3], [4], accuracy [5], and reproducibility [6], a better visual outcome [7], [8], and less epithelial ingrowth [9] and dry eye complications [10]. The applications of FSLs for therapeutic corneal surgeries, on the other hand, have lagged behind [11]. Yet, expectations remain high [12]–[14], as a significant percentage of the corneal transplants (46 196 lamellar and penetrating corneal transplants reported in 2011 by the Eye Bank Association of America for the United States only [15]) could potentially benefit from the high precision FSL technology. Femtosecond lasers have been proposed for the dissection of the donor button and/or recipient cornea in corneal endothelial keratoplasty [16] and in deep anterior lamellar keratoplasty [17], [18] and they are now used to improve the design of the penetrating keratoplasty wound [19], [20]. However, while the FSL parameters seem to be well mastered for an anterior ablation in normal clear corneas, the interaction between FSL pulses and a diseased and hydrated corneal tissue still remains to be understood. Furthermore, a precise control of this interaction will be essential to allow better surgical results and acceptance by corneal surgeons worldwide.

The goal of this study was to evaluate the effect of corneal hydration on the quality of the FSL lamellar cut, all parameters being otherwise controlled and similar. All ablations were performed at an intended depth of 220 µm, which corresponded to the limit of the depth range that the LASIK FSLs can typically operate. The outcome parameters analyzed were the difference between intended and achieved ablation depth, ease of flap separation, and smoothness of the ablated surface. Ex-vivo human eye bank eyes were used.

## Methods

### Preoperative globe preparation

Twenty-two human donor eyes unsuitable for transplantation were obtained from the Quebec Eye Bank (Montreal, QC, Canada). Eyes with slit lamp signs of previous eye surgery or inflammation (including corneal opacity and vascularization) were excluded from the study.

According to the initial pachymetry, eyes were either directly assigned to laser ablation, or subjected to induced stromal hydration by leaving them in a humid chamber at 4°C until the required central corneal thickness (CCT) was reached, based on the temperature reversal phenomenon [21]. A non or minimally hydrated cornea with a preoperative thickness within the normal range [22], [23] was used as the reference. This cornea was also meant to illustrate the typical ablation surface obtained after FSL dissection of a LASIK flap in a normal eye.

Laser surgery was performed within 48 hours of death. The loosely adherent corneal epithelium was gently scraped off with a microsponge. A hand held tonometer (Tonovet TV01, Tiolat Oy, Helsinki, Finland) was used to monitor intraocular pressure (IOP). The IOP was maintained between 18 to 22 mm Hg during the experimentation by injection of balanced salt solution (BSS, Alcon, Mississauga, Ontario, Canada) in the vitreous through the optic nerve stump to eliminate possible effects of IOP changes on corneal thickness and curvature. The eye was placed in an eye bank plastic holder with a 14 mm central circular front opening. The preoperative level of corneal hydration was confirmed by measuring CCT by ultrasound pachymetry (DGH Technology, Exton, PA, USA) and optical coherence tomography (OCT) (Visante, Carl Zeiss Meditec, Inc., Jena, Germany). Both methods being in agreement (ICC = 0.900, 95% CI = 0.778–0.957, p<0.0001), only the OCT was used after laser ablation, since intrastromal cavitation bubbles interfered with ultrasound pachymetry.

### FSL surgical procedure

A commercially available FSL (Visumax FSL system, Carl Zeiss Meditec, Inc., Germany) was used to cut the anterior corneal flap. The laser parameters were: wavelength 1043 nm, pulse frequency 250 kHz, pulse duration 180–200 fs, and a power per pulse of 150 nJ. The eye was centered, the surface moistened with BSS, and the anterior corneal surface was applanated with the disposable interface lens. Concavity of this applanation lens minimized distortion of the corneal surface during the ablation. The flap diameter was 8 mm and the intended depth was 220 µm from the corneal surface, which corresponded to the maximum ablation depth available on this FSL commercial unit. A continuous spiral out-in pattern of cavitation bubbles was applied and duration of ablation was 19 seconds, including the side cut and superior hinge dissection.

### Assessment of the laser cut

#### Achieved ablation depth

**Figure 1:**
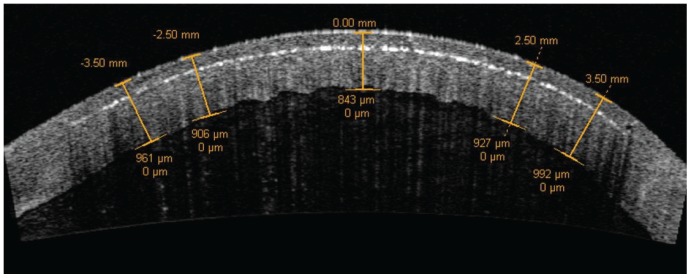
Achieved ablation depth. Representative OCT image taken after the laser cut. Ablation depth was measured in the central (0.00 mm), intermediate (±2.50 mm) and mid-peripheral (±3.50 mm) cornea. The middle of the cavitation bubble layer was considered for the identification of the ablation depth.

Optical coherence tomography was repeated immediately after FSL ablation and images were saved using the enhanced high-resolution horizontal corneal scan protocol provided by the Visante software (**Figure 1**). The ImageJ software (ImageJ 1.44, National Institute of Health, Bethesda, MD) [24] was used by 2 independent observers to measure the flap thickness on the OCT images in the central (0.00 mm), intermediate (2.50 mm from the center on each side) and mid-peripheral (3.50 mm from the center on each side) cornea. A strong inter-observer correlation was found for the ablation depth measurements in all corneal positions, with ICC values ranging from 0.771 to 0.945 (p<0.0001), and the mean of the two observers' values was used for each measurement. The middle of the cavitation bubble layer was considered for the identification of the ablation depth.

#### Flap separability

**Figure 2:**
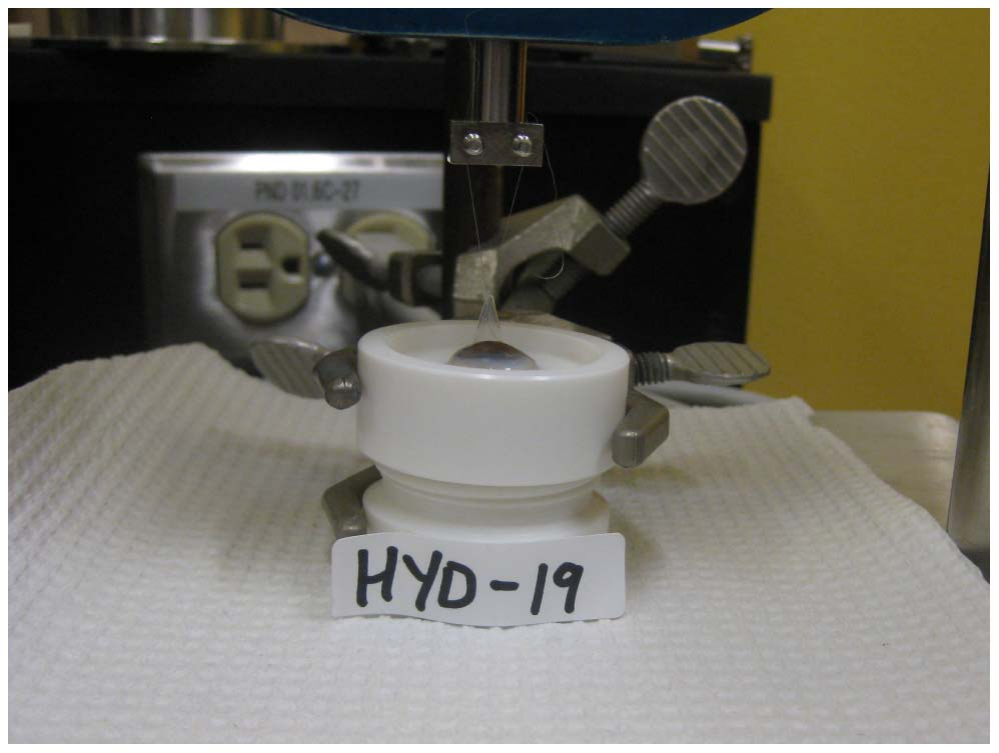
Flap separability testing. The flap was attached to the mechanical tester and lifted until the hinge was reached. Flap separability was assessed by measuring the mean tensile force generated to separate the flap from the posterior part of the cornea.

Flap separability after the cut was assessed by measuring the mean force generated during the separation of the flap from the posterior part of the cornea. A full thickness 8.0 suture (Biosorb C3, Alcon lab Inc., Fortworth, TX, USA) was inserted through the edge of the flap, opposite to the midpoint of the hinge. This suture was attached to a micromechanical testing system (Mach-1 A300, Biomomentum, Laval, QC) (**Figure 2**) composed of an actuator that controls the displacement and a load cell that measures the force. In all cases, a vertical displacement at constant speed (0.5 mm/s) was applied to lift the flap until pulling on the hinge. The tensile force generated during the displacement was recorded every 100 ms. The mean tensile force was proportional to the energy required to break the tissue bridges and separate the flap, and was used to characterize flap separability. An easy separation, represented by a low tensile force, indicated a lamellar cut with minimal or no residual tissue bridges.

#### Quality of the ablated stromal surface

The corneoscleral buttons were then dissected and the flap gently cut off. The specimens were fixed in formaldehyde 10% and processed for scanning electron microscopy (SEM). Briefly, washing in BSS for 5 minutes was followed by dehydration in ascending concentrations of ethanol (25%, 50%, 75% and 100%, ten minutes each), followed by infiltration with three changes of hexamethyldisilazane (HMDS) each lasting for 10 minutes. The samples were allowed to slowly air-dry overnight. They were then coated with 20 nm of gold by resistive thermal evaporation and examined (JEOL JSM-6300F, Tokyo, Japan) at 5 kV and 10 mm working distance. SEM photos were taken at 1000× magnification, at three different locations within the ablated bed.

Smoothness of the ablated bed was then evaluated based on the texture analysis of the SEM images [2]. Image texture, defined as variations in the pixel intensities (variations in the gray-levels), was analyzed using the Haralick texture contrast parameter [25]. The usefulness of this parameter has been well documented in biomedical sciences [26], and more specifically, for the quantification of corneal stromal surface roughness as observed on a SEM image [27]. The Haralick contrast parameter was computed for each SEM image (Matlab R2009b version). Haralick contrast values increase with roughness.

### Statistical analysis

The Spearman's rank correlation coefficient (r) was calculated to study the association between the degree of corneal hydration, as measured by the preoperative CCT, and the outcome parameters (achieved ablation depth, mean tensile force measured during flap detachment, and Haralick surface roughness index). Inter-observer reproducibility for the pachymetry measurements made on the OCT images using ImageJ was assessed using intra-class correlation coefficients (ICCs). Intra-class correlation coefficients were also used to assess inter-instrument reproducibility for the corneal thickness measurements performed by ultrasound pachymetry vs. OCT. A p value of less than 0.05 was considered to be statistically significant. Analyses were conducted with the SPSS software (Version 19, SPSS, Inc., Chicago, IL).

## Results

### Level of corneal hydration

Twenty-two corneas with variable levels of hydration were included in this study. The preoperative central pachymetry ranged from 547 to 1104 µm (mean ± SEM: 833±30 µm).

### Achieved ablation depth

**Figure 3:**
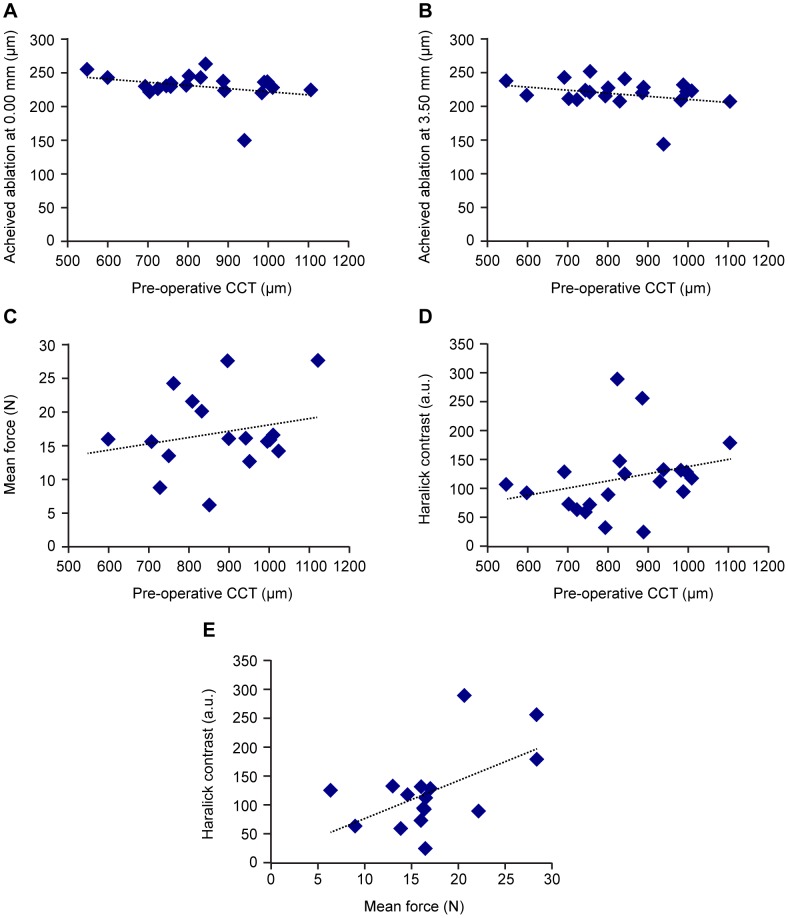
Quality of the laser cut. Effect of corneal hydration on the achieved ablation depth in the central and peripheral cornea (A and B), flap separability (C) and surface roughness (D). Surface roughness as a function of flap separability (E) is also illustrated.

In **Figures 3A and 3B**, the ablation depth in the central (0.00 mm) and mid-peripheral (±3.50 mm away from the center) cornea is plotted as a function of preoperative CCT. A negative correlation was found between the achieved ablation depth in the mid-peripheral cornea and the level of corneal hydration (r = −0.626, p = 0.003), the ablation being more superficial in more edematous corneas. The negative correlations also observed between the level of corneal hydration and the ablation depth achieved in the central and intermediate positions did not reach statistical significance (at 0.00 mm: r = −0.245, p = 0.298 and at ±2.50 mm: r = −0.244, p = 0.301). All these measurements, however, were significantly inter-correlated (±2.50 mm vs. 0.00 mm: r = 0.474, p = 0.035 and ±2.50 mm vs. ±3.50 mm: r = 0.541, p = 0.014), which allows to think that the same effect of hydration on the ablation depth could also exist at 0.00 mm and ±2.50 mm.

### Flap separability

In **Figure 3C**, the mean tensile force measured during detachment of the corneal flap from the stromal bed was plotted as a function of preoperative CCT. No significant correlation was found between the force required for separation and the level of corneal hydration (r = 0.189, p = 0.468). Similarly, no correlations were found between the tensile force and the ablation depth achieved either in the center (r = 0.196; p = 0.483) or ±3.50 mm away from the center (r = −0.082; p = 0.771).

### Quality of the ablated stromal surface

The positive correlation observed between the Haralick contrast (index of surface roughness of the stromal bed after lifting the corneal flap) and the level of the corneal hydration tended to reach but did not reach statistical significance (r = 0.416, p = 0.061) (**Figure 3D**), meaning that laser ablation in more edematous corneas tended to result in rougher stromal surfaces. The Haralick contrast also seemed to increase as a function of the mean tensile force generated to lift the flap (**Figure 3E**), the corneal bed surface being rougher when the flap was more difficult to lift, but this tendency did not reach statistical significance either (r = 0.374, p = 0.154).

**Figure 4:**
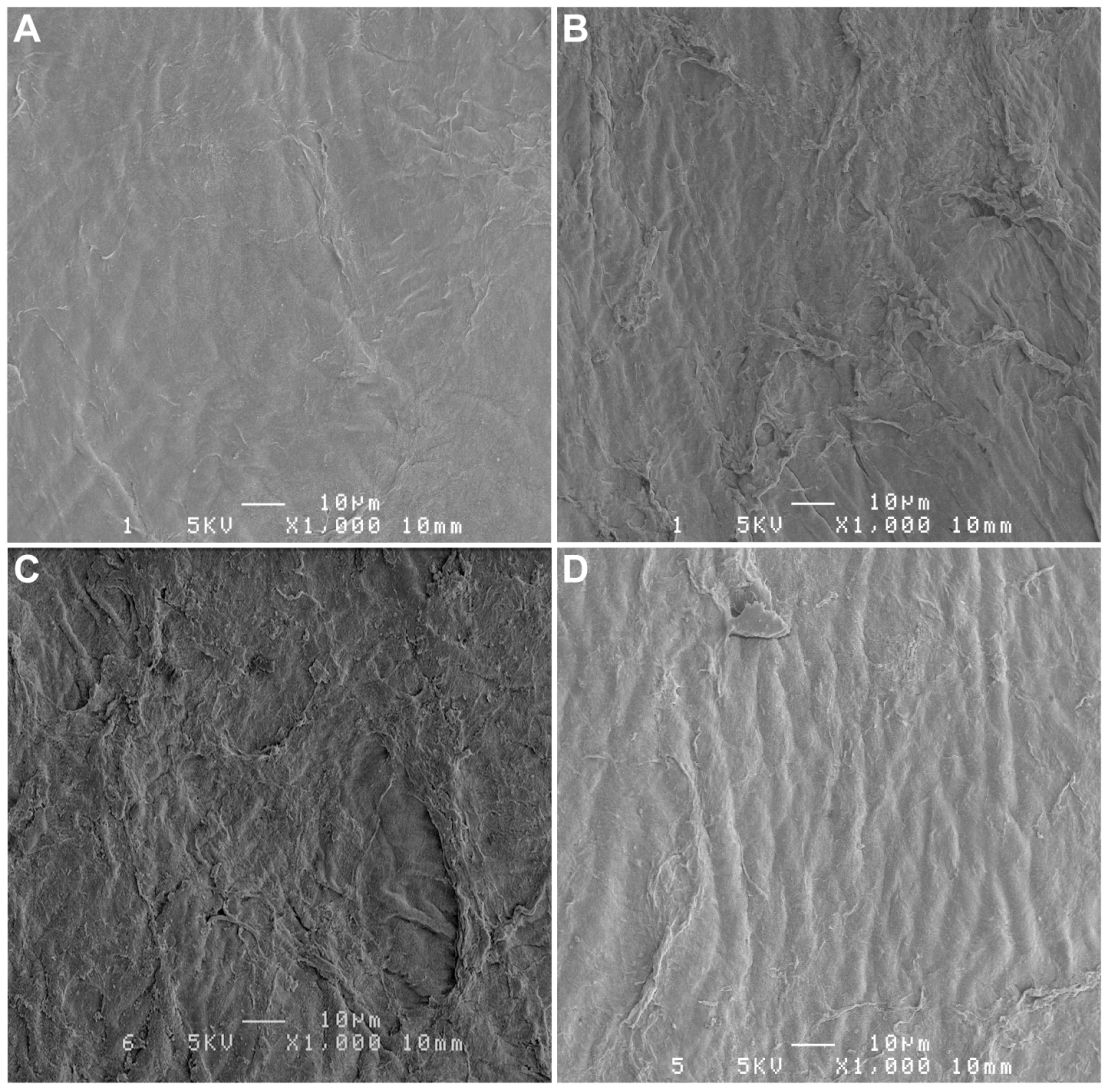
Representative scanning electron microscopy images of the post-ablation stromal surface for various levels of preoperative corneal hydration. CCT = 598 µm (A); CCT = 801 µm (B); CCT = 996 µm (C); and CCT = 1104 µm (D). These images illustrate the difficulty of quantifying corneal surface roughness based on human eye perception. The cornea illustrated in (A) had a preoperative thickness within the normal range and was used as a reference.

**Figure 4A-D** illustrates different degrees of surface roughness for levels of hydration ranging from 598 µm (**Figure 4A**) to 1104 µm (**Figure 4D**). It demonstrates well the difficulty of quantifying surface roughness based on subjective observation. The cornea shown in **Figure 4A** had a preoperative thickness within the normal range and can be used as a reference. It also represented the typical example of the type of surface obtained after FSL dissection of a LASIK flap in a normal eye.

## Discussion

To the best of our knowledge, this is the first time the effect of corneal hydration on the quality of the FSL anterior lamellar cut is studied. To insure clinical relevance, a commercially available FSL designed for ophthalmic surgery was used.

The negative correlation found between the achieved ablation depth in the mid-peripheral cornea and the level of corneal hydration indicated that the ablation was more superficial in thicker corneas. Even if not statistically significant, the same pattern was observed at 0.00 mm and ±2.50 mm. These results support the idea that as the level of corneal hydration increases, the FSL ablation becomes more superficial and less precise, leaving residual tissue bridges, which necessitate a higher force to separate the flap, and resulting in a rougher stromal bed surface. A sample size of 123 corneas would have been necessary to confirm the observed correlation of −0.245 between the level of corneal hydration and the achieved central ablation depth with a power of 80% at a significance level of 0.05. Similarly, 42 corneas would have allowed to detect a significant correlation of 0.416 between the level of corneal hydration and the Haralick contrast with the same precision.

These findings are supported by theory. Corneal transparency depends on the uniform arrangement of the collagen fibers, which are the main structural components of the corneal stroma [28]. In an edematous cornea, the collagen fibers arrangement is perturbed, the fibrils density decreases, and “lakes” appear in the fibril-free regions [28]–[30], resulting in an increased mismatch between the collagen fibrils and the extrafibrillar material refractive indices [31], [32]. All these phenomena lead to increased light scattering, which results in a transverse broadening and a longitudinal decrease of the laser intensity profiles with the propagation distance. As a consequence, the ablation spot is closer to the surface (**Figures 3A and 3B**), has a lower intensity and a larger size, producing a lower quality cut (**Figure 3D**).

Light scattering is also expected to be more pronounced in the edematous posterior stroma because of the increased swelling properties of the posterior corneal layers, which could be explained by the tissue ultrastructure and by the distribution of the mechanical and biochemical properties across the cornea [33], [34].

Literature review confirms that although FSLs have allowed major improvements in corneal surgery, commercially available systems designed for ophthalmology use are not yet adapted for ablation in edematous corneas. Deep ablations in hydrated corneas are especially challenging for the following reasons: (1) Accuracy and predictability of the FSL cut decreases for deep ablations [35], [36]. FSL deep lamellar ablations result in residual tissue bridges and increased surface roughness [35]–[37], which may necessitate phototherapeutic resurfacing [38] or multipass ablations [39], [40]. (2) In edematous corneas, such as eye bank donor corneas, scattering impairs deep ablation. Attenuation of the FSL beam necessitates increased levels of energy, with associated risk for increased collateral damage to the endothelium [16], [41]. One would have to evaluate corneal transparency before adjusting the laser energy. (3) Deep lamellar dissections in edematous corneas may require ablation depths up to 800 µm or more, while the ablation depth of commercially available FSL systems is often limited (the range of this limitation varying with the FSL brand). (4) The flat lamellar cuts (horizontal plane) produced by the commercially available FSL systems yield posterior lamellar grafts thinner in the center and thicker in the periphery [42], which is known to induce a hyperopic shift in the operated eye. Customized deep ablation profiles adapted to the aspherical corneal curvature would be needed. (5) The flat applanation of the curved cornea used to simplify the optical setup and stabilize the tissue during ablation induces concentric folds in the more loosely arranged posterior stromal collagen fibers, resulting in concentric circular ridges on the surface of the ablated posterior graft [36]. In severely hydrated corneas, the uneven distribution of the edema, which accumulates preferentially in the central posterior stroma, may accentuate this concentric pattern [33], [43]. (6) And finally, ablation of an edematous cornea necessitates removal of the damaged and/or microcystic epithelium to minimize imprecision of the cut, a step that would rather be avoided as it increases postoperative discomfort and risk for infection.

The results of this study support the hypothesis that the quality of the FSL lamellar cut decreases as the level corneal hydration increases. These results are corroborated by theory and clinical observations by others. These considerations are highly clinically relevant considering the expectations, the potential for improvement of corneal surgery in diseased corneas, and the risk for complications if the achieved FSL ablation does not reach the expected standards of quality. The highly sophisticated wound profiles being developed for full thickness corneal transplantation (zigzag, top hat and mushroom-style incisions) [44]. for instance, would not be conceivable in edematous corneas if the ablation depth and laser spot size and intensity could not be relied on. Inadequate architecture of these complex wounds could result in misalignment, wound leak, wound instability and severe and unpredictable astigmatism. Similarly, preparation of a thin posterior corneal flap for endothelial keratoplasty using a FSL would require the highest precision to avoid irreversible damage to the endothelial cells, eye bank corneas being generally somewhat hydrated at the time of processing [45].

Although FSL is still considered in the field as the tool of the future for lamellar corneal dissection, optimization of the laser parameters, a better definition of the limits of this tool and a better understanding of FSL-tissue interaction in the posterior layers of the edematous cornea will be needed to improve the safety and outcomes of therapeutic applications in hydrated corneas.
